# Supplementary material for: Baseline cognition and stimulation site shape the effects of tdcs on verbal fluency in older and younger adults
Source: GeroScience. 2026 Mar 23;48(4):5161–74. doi: 10.1007/s11357-026-02104-2 (PMC13575039; doi:10.1007/s11357-026-02104-2)
Supplement: Supplementary file 1 [file 11357_2026_2104_MOESM1_ESM.docx]

**Supplementary Table 1. Complete omnibus ANOVA results for all main effects and interactions.**

| **Within Subjects Effects** | | | | | | | | | | | | | |
| --- | --- | --- | --- | --- | --- | --- | --- | --- | --- | --- | --- | --- | --- |
| **Cases** | | **Sum of Squares** | | **df** | | **Mean Square** | | **F** | | **p** | | **η²_p_** | |
| Stimulation Type |  | 9.389 |  | 1 |  | 9.389 |  | 0.665 |  | 0.416 |  | 0.005 |  |
| Stimulation Type ✻ Region |  | 15.125 |  | 1 |  | 15.125 |  | 1.072 |  | 0.302 |  | 0.008 |  |
| Stimulation Type ✻ AGEGROUP |  | 0.281 |  | 1 |  | 0.281 |  | 0.020 |  | 0.888 |  | 1.423×10^-4^ |  |
| Stimulation Type ✻ Region ✻ AGEGROUP |  | 10.503 |  | 1 |  | 10.503 |  | 0.744 |  | 0.390 |  | 0.005 |  |
| Residuals |  | 1976.201 |  | 140 |  | 14.116 |  |  |  |  |  |  |  |
| Fluency Type |  | 7917.014 |  | 1 |  | 7917.014 |  | 301.148 |  | < .001 |  | 0.683 |  |
| Fluency Type ✻ Region |  | 3.125 |  | 1 |  | 3.125 |  | 0.119 |  | 0.731 |  | 8.483×10^-4^ |  |
| Fluency Type ✻ AGEGROUP |  | 375.837 |  | 1 |  | 375.837 |  | 14.296 |  | < .001 |  | 0.093 |  |
| Fluency Type ✻ Region ✻ AGEGROUP |  | 0.003 |  | 1 |  | 0.003 |  | 1.321×10^-4^ |  | 0.991 |  | 9.434×10^-7^ |  |
| Residuals |  | 3680.521 |  | 140 |  | 26.289 |  |  |  |  |  |  |  |
| Time |  | 7750.125 |  | 1 |  | 7750.125 |  | 335.811 |  | < .001 |  | 0.706 |  |
| Time ✻ Region |  | 37.556 |  | 1 |  | 37.556 |  | 1.627 |  | 0.204 |  | 0.011 |  |
| Time ✻ AGEGROUP |  | 294.031 |  | 1 |  | 294.031 |  | 12.740 |  | < .001 |  | 0.083 |  |
| Time ✻ Region ✻ AGEGROUP |  | 39.753 |  | 1 |  | 39.753 |  | 1.723 |  | 0.192 |  | 0.012 |  |
| Residuals |  | 3231.035 |  | 140 |  | 23.079 |  |  |  |  |  |  |  |
| Stimulation Type ✻ Fluency Type |  | 7.031 |  | 1 |  | 7.031 |  | 0.617 |  | 0.433 |  | 0.004 |  |
| Stimulation Type ✻ Fluency Type ✻ Region |  | 77.087 |  | 1 |  | 77.087 |  | 6.766 |  | 0.010 |  | 0.046 |  |
| Stimulation Type ✻ Fluency Type ✻ AGEGROUP |  | 24.500 |  | 1 |  | 24.500 |  | 2.150 |  | 0.145 |  | 0.015 |  |
| Stimulation Type ✻ Fluency Type ✻ Region ✻ AGEGROUP |  | 7.347 |  | 1 |  | 7.347 |  | 0.645 |  | 0.423 |  | 0.005 |  |
| Residuals |  | 1595.035 |  | 140 |  | 11.393 |  |  |  |  |  |  |  |
| Stimulation Type ✻ Time |  | 2.920 |  | 1 |  | 2.920 |  | 0.315 |  | 0.575 |  | 0.002 |  |
| Stimulation Type ✻ Time ✻ Region |  | 45.920 |  | 1 |  | 45.920 |  | 4.957 |  | 0.028 |  | 0.034 |  |
| Stimulation Type ✻ Time ✻ AGEGROUP |  | 3.125 |  | 1 |  | 3.125 |  | 0.337 |  | 0.562 |  | 0.002 |  |
| Stimulation Type ✻ Time ✻ Region ✻ AGEGROUP |  | 0.125 |  | 1 |  | 0.125 |  | 0.013 |  | 0.908 |  | 9.637×10^-5^ |  |
| Residuals |  | 1296.910 |  | 140 |  | 9.264 |  |  |  |  |  |  |  |
| Fluency Type ✻ Time |  | 218.753 |  | 1 |  | 218.753 |  | 13.023 |  | < .001 |  | 0.085 |  |
| Fluency Type ✻ Time ✻ Region |  | 0.087 |  | 1 |  | 0.087 |  | 0.005 |  | 0.943 |  | 3.691×10^-5^ |  |
| Fluency Type ✻ Time ✻ AGEGROUP |  | 0.889 |  | 1 |  | 0.889 |  | 0.053 |  | 0.818 |  | 3.779×10^-4^ |  |
| Fluency Type ✻ Time ✻ Region ✻ AGEGROUP |  | 11.681 |  | 1 |  | 11.681 |  | 0.695 |  | 0.406 |  | 0.005 |  |
| Residuals |  | 2351.590 |  | 140 |  | 16.797 |  |  |  |  |  |  |  |
| Stimulation Type ✻ Fluency Type ✻ Time |  | 0.125 |  | 1 |  | 0.125 |  | 0.012 |  | 0.914 |  | 8.427×10^-5^ |  |
| Stimulation Type ✻ Fluency Type ✻ Time ✻ Region |  | 36.125 |  | 1 |  | 36.125 |  | 3.410 |  | 0.067 |  | 0.024 |  |
| Stimulation Type ✻ Fluency Type ✻ Time ✻ AGEGROUP |  | 59.587 |  | 1 |  | 59.587 |  | 5.625 |  | 0.019 |  | 0.039 |  |
| Stimulation Type ✻ Fluency Type ✻ Time ✻ Region ✻ AGEGROUP |  | 2.531 |  | 1 |  | 2.531 |  | 0.239 |  | 0.626 |  | 0.002 |  |
| Residuals |  | 1483.132 |  | 140 |  | 10.594 |  |  |  |  |  |  |  |
|  | | | | | | | | | | | | | |
| *Note.*  Type III Sum of Squares | | | | | | | | | | | | | |

| **Between Subjects Effects** | | | | | | | | | | | | | |
| --- | --- | --- | --- | --- | --- | --- | --- | --- | --- | --- | --- | --- | --- |
| **Cases** | | **Sum of Squares** | | **df** | | **Mean Square** | | **F** | | **p** | | **η²_p_** | |
| Region |  | 28.753 |  | 1 |  | 28.753 |  | 0.394 |  | 0.531 |  | 0.003 |  |
| AGEGROUP |  | 355.556 |  | 1 |  | 355.556 |  | 4.867 |  | 0.029 |  | 0.034 |  |
| Region ✻ AGEGROUP |  | 26.889 |  | 1 |  | 26.889 |  | 0.368 |  | 0.545 |  | 0.003 |  |
| Residuals |  | 10227.521 |  | 140 |  | 73.054 |  |  |  |  |  |  |  |
|  | | | | | | | | | | | | | |
| *Note.*  Type III Sum of Squares | | | | | | | | | | | | | |

**Descriptives**

| **Descriptives** | | | | | | | | | | | | | | | | | | | |
| --- | --- | --- | --- | --- | --- | --- | --- | --- | --- | --- | --- | --- | --- | --- | --- | --- | --- | --- | --- |
| **Stimulation Type** | | **Fluency Type** | | **Time** | | **Region** | | **AGEGROUP** | | **N** | | **Mean** | | **SD** | | **SE** | | **Coefficient of variation** | |
| Sham |  | Semantic |  | F15 |  | F |  | Older |  | 36 |  | 15.667 |  | 3.234 |  | 0.539 |  | 0.206 |  |
|  |  |  |  |  |  |  |  | Younger |  | 36 |  | 16.972 |  | 3.460 |  | 0.577 |  | 0.204 |  |
|  |  |  |  |  |  | T |  | Older |  | 36 |  | 15.583 |  | 3.237 |  | 0.540 |  | 0.208 |  |
|  |  |  |  |  |  |  |  | Younger |  | 36 |  | 16.194 |  | 2.896 |  | 0.483 |  | 0.179 |  |
|  |  |  |  | L45 |  | F |  | Older |  | 36 |  | 21.056 |  | 5.356 |  | 0.893 |  | 0.254 |  |
|  |  |  |  |  |  |  |  | Younger |  | 36 |  | 19.778 |  | 7.059 |  | 1.177 |  | 0.357 |  |
|  |  |  |  |  |  | T |  | Older |  | 36 |  | 20.194 |  | 5.549 |  | 0.925 |  | 0.275 |  |
|  |  |  |  |  |  |  |  | Younger |  | 36 |  | 20.972 |  | 5.639 |  | 0.940 |  | 0.269 |  |
|  |  | Phonemic |  | F15 |  | F |  | Older |  | 36 |  | 11.139 |  | 3.053 |  | 0.509 |  | 0.274 |  |
|  |  |  |  |  |  |  |  | Younger |  | 36 |  | 9.694 |  | 3.179 |  | 0.530 |  | 0.328 |  |
|  |  |  |  |  |  | T |  | Older |  | 36 |  | 10.000 |  | 2.918 |  | 0.486 |  | 0.292 |  |
|  |  |  |  |  |  |  |  | Younger |  | 36 |  | 9.667 |  | 3.610 |  | 0.602 |  | 0.373 |  |
|  |  |  |  | L45 |  | F |  | Older |  | 36 |  | 19.417 |  | 7.439 |  | 1.240 |  | 0.383 |  |
|  |  |  |  |  |  |  |  | Younger |  | 36 |  | 14.528 |  | 4.754 |  | 0.792 |  | 0.327 |  |
|  |  |  |  |  |  | T |  | Older |  | 36 |  | 17.333 |  | 5.550 |  | 0.925 |  | 0.320 |  |
|  |  |  |  |  |  |  |  | Younger |  | 36 |  | 13.944 |  | 5.722 |  | 0.954 |  | 0.410 |  |
| Anodal |  | Semantic |  | F15 |  | F |  | Older |  | 36 |  | 16.472 |  | 3.334 |  | 0.556 |  | 0.202 |  |
|  |  |  |  |  |  |  |  | Younger |  | 36 |  | 17.667 |  | 4.216 |  | 0.703 |  | 0.239 |  |
|  |  |  |  |  |  | T |  | Older |  | 36 |  | 15.556 |  | 3.367 |  | 0.561 |  | 0.216 |  |
|  |  |  |  |  |  |  |  | Younger |  | 36 |  | 16.389 |  | 2.821 |  | 0.470 |  | 0.172 |  |
|  |  |  |  | L45 |  | F |  | Older |  | 36 |  | 22.083 |  | 6.733 |  | 1.122 |  | 0.305 |  |
|  |  |  |  |  |  |  |  | Younger |  | 36 |  | 19.750 |  | 6.063 |  | 1.011 |  | 0.307 |  |
|  |  |  |  |  |  | T |  | Older |  | 36 |  | 21.028 |  | 7.033 |  | 1.172 |  | 0.334 |  |
|  |  |  |  |  |  |  |  | Younger |  | 36 |  | 20.167 |  | 6.349 |  | 1.058 |  | 0.315 |  |
|  |  | Phonemic |  | F15 |  | F |  | Older |  | 36 |  | 11.167 |  | 2.823 |  | 0.471 |  | 0.253 |  |
|  |  |  |  |  |  |  |  | Younger |  | 36 |  | 9.972 |  | 3.167 |  | 0.528 |  | 0.318 |  |
|  |  |  |  |  |  | T |  | Older |  | 36 |  | 10.861 |  | 2.664 |  | 0.444 |  | 0.245 |  |
|  |  |  |  |  |  |  |  | Younger |  | 36 |  | 9.083 |  | 2.999 |  | 0.500 |  | 0.330 |  |
|  |  |  |  | L45 |  | F |  | Older |  | 36 |  | 16.722 |  | 6.700 |  | 1.117 |  | 0.401 |  |
|  |  |  |  |  |  |  |  | Younger |  | 36 |  | 14.028 |  | 5.135 |  | 0.856 |  | 0.366 |  |
|  |  |  |  |  |  | T |  | Older |  | 36 |  | 18.194 |  | 4.387 |  | 0.731 |  | 0.241 |  |
|  |  |  |  |  |  |  |  | Younger |  | 36 |  | 15.889 |  | 5.371 |  | 0.895 |  | 0.338 |  |
|  | | | | | | | | | | | | | | | | | | | |

**Supplementary Table 2. Effects of fluid intelligence (MaRs-IB test) on verbal fluency.**

| **Within Subjects Effects** | | | | | | | | | | | | | |
| --- | --- | --- | --- | --- | --- | --- | --- | --- | --- | --- | --- | --- | --- |
| **Cases** | | **Sum of Squares** | | **df** | | **Mean Square** | | **F** | | **p** | | **η²_p_** | |
| Stimulation Type |  | 21.909 |  | 1 |  | 21.909 |  | 1.603 |  | 0.208 |  | 0.011 |  |
| Stimulation Type ✻ Region |  | 84.301 |  | 1 |  | 84.301 |  | 6.167 |  | 0.014 |  | 0.043 |  |
| Stimulation Type ✻ AGEGROUP |  | 0.636 |  | 1 |  | 0.636 |  | 0.047 |  | 0.830 |  | 3.372×10^-4^ |  |
| Stimulation Type ✻ MARS Test |  | 17.156 |  | 1 |  | 17.156 |  | 1.255 |  | 0.265 |  | 0.009 |  |
| Stimulation Type ✻ Region ✻ MARS Test |  | 73.745 |  | 1 |  | 73.745 |  | 5.395 |  | 0.022 |  | 0.038 |  |
| Stimulation Type ✻ Region ✻ AGEGROUP |  | 0.160 |  | 1 |  | 0.160 |  | 0.012 |  | 0.914 |  | 8.458×10^-5^ |  |
| Residuals |  | 1886.463 |  | 138 |  | 13.670 |  |  |  |  |  |  |  |
| Fluency Type |  | 247.037 |  | 1 |  | 247.037 |  | 9.272 |  | 0.003 |  | 0.063 |  |
| Fluency Type ✻ Region |  | 3.834 |  | 1 |  | 3.834 |  | 0.144 |  | 0.705 |  | 0.001 |  |
| Fluency Type ✻ AGEGROUP |  | 328.546 |  | 1 |  | 328.546 |  | 12.331 |  | < .001 |  | 0.082 |  |
| Fluency Type ✻ MARS Test |  | 0.891 |  | 1 |  | 0.891 |  | 0.033 |  | 0.855 |  | 2.423×10^-4^ |  |
| Fluency Type ✻ Region ✻ MARS Test |  | 2.730 |  | 1 |  | 2.730 |  | 0.102 |  | 0.749 |  | 7.420×10^-4^ |  |
| Fluency Type ✻ Region ✻ AGEGROUP |  | 0.212 |  | 1 |  | 0.212 |  | 0.008 |  | 0.929 |  | 5.752×10^-5^ |  |
| Residuals |  | 3676.950 |  | 138 |  | 26.645 |  |  |  |  |  |  |  |
| Time |  | 39.360 |  | 1 |  | 39.360 |  | 1.740 |  | 0.189 |  | 0.012 |  |
| Time ✻ Region |  | 5.515 |  | 1 |  | 5.515 |  | 0.244 |  | 0.622 |  | 0.002 |  |
| Time ✻ AGEGROUP |  | 381.393 |  | 1 |  | 381.393 |  | 16.864 |  | < .001 |  | 0.109 |  |
| Time ✻ MARS Test |  | 107.997 |  | 1 |  | 107.997 |  | 4.775 |  | 0.031 |  | 0.033 |  |
| Time ✻ Region ✻ MARS Test |  | 1.615 |  | 1 |  | 1.615 |  | 0.071 |  | 0.790 |  | 5.173×10^-4^ |  |
| Time ✻ Region ✻ AGEGROUP |  | 40.618 |  | 1 |  | 40.618 |  | 1.796 |  | 0.182 |  | 0.013 |  |
| Residuals |  | 3120.951 |  | 138 |  | 22.616 |  |  |  |  |  |  |  |
| Stimulation Type ✻ Fluency Type |  | 0.370 |  | 1 |  | 0.370 |  | 0.032 |  | 0.858 |  | 2.329×10^-4^ |  |
| Stimulation Type ✻ Fluency Type ✻ Region |  | 19.361 |  | 1 |  | 19.361 |  | 1.683 |  | 0.197 |  | 0.012 |  |
| Stimulation Type ✻ Fluency Type ✻ AGEGROUP |  | 21.789 |  | 1 |  | 21.789 |  | 1.895 |  | 0.171 |  | 0.014 |  |
| Stimulation Type ✻ Fluency Type ✻ MARS Test |  | 0.016 |  | 1 |  | 0.016 |  | 0.001 |  | 0.970 |  | 1.011×10^-5^ |  |
| Stimulation Type ✻ Fluency Type ✻ Region ✻ MARS Test |  | 7.881 |  | 1 |  | 7.881 |  | 0.685 |  | 0.409 |  | 0.005 |  |
| Stimulation Type ✻ Fluency Type ✻ Region ✻ AGEGROUP |  | 2.895 |  | 1 |  | 2.895 |  | 0.252 |  | 0.617 |  | 0.002 |  |
| Residuals |  | 1587.123 |  | 138 |  | 11.501 |  |  |  |  |  |  |  |
| Stimulation Type ✻ Time |  | 6.927 |  | 1 |  | 6.927 |  | 0.755 |  | 0.386 |  | 0.005 |  |
| Stimulation Type ✻ Time ✻ Region |  | 35.684 |  | 1 |  | 35.684 |  | 3.891 |  | 0.051 |  | 0.027 |  |
| Stimulation Type ✻ Time ✻ AGEGROUP |  | 6.840 |  | 1 |  | 6.840 |  | 0.746 |  | 0.389 |  | 0.005 |  |
| Stimulation Type ✻ Time ✻ MARS Test |  | 8.899 |  | 1 |  | 8.899 |  | 0.970 |  | 0.326 |  | 0.007 |  |
| Stimulation Type ✻ Time ✻ Region ✻ MARS Test |  | 22.817 |  | 1 |  | 22.817 |  | 2.488 |  | 0.117 |  | 0.018 |  |
| Stimulation Type ✻ Time ✻ Region ✻ AGEGROUP |  | 1.336 |  | 1 |  | 1.336 |  | 0.146 |  | 0.703 |  | 0.001 |  |
| Residuals |  | 1265.661 |  | 138 |  | 9.171 |  |  |  |  |  |  |  |
| Fluency Type ✻ Time |  | 18.798 |  | 1 |  | 18.798 |  | 1.106 |  | 0.295 |  | 0.008 |  |
| Fluency Type ✻ Time ✻ Region |  | 3.016 |  | 1 |  | 3.016 |  | 0.177 |  | 0.674 |  | 0.001 |  |
| Fluency Type ✻ Time ✻ AGEGROUP |  | 0.159 |  | 1 |  | 0.159 |  | 0.009 |  | 0.923 |  | 6.784×10^-5^ |  |
| Fluency Type ✻ Time ✻ MARS Test |  | 2.522 |  | 1 |  | 2.522 |  | 0.148 |  | 0.701 |  | 0.001 |  |
| Fluency Type ✻ Time ✻ Region ✻ MARS Test |  | 2.906 |  | 1 |  | 2.906 |  | 0.171 |  | 0.680 |  | 0.001 |  |
| Fluency Type ✻ Time ✻ Region ✻ AGEGROUP |  | 7.369 |  | 1 |  | 7.369 |  | 0.433 |  | 0.511 |  | 0.003 |  |
| Residuals |  | 2346.251 |  | 138 |  | 17.002 |  |  |  |  |  |  |  |
| Stimulation Type ✻ Fluency Type ✻ Time |  | 16.480 |  | 1 |  | 16.480 |  | 1.553 |  | 0.215 |  | 0.011 |  |
| Stimulation Type ✻ Fluency Type ✻ Time ✻ Region |  | 0.087 |  | 1 |  | 0.087 |  | 0.008 |  | 0.928 |  | 5.914×10^-5^ |  |
| Stimulation Type ✻ Fluency Type ✻ Time ✻ AGEGROUP |  | 36.834 |  | 1 |  | 36.834 |  | 3.470 |  | 0.065 |  | 0.025 |  |
| Stimulation Type ✻ Fluency Type ✻ Time ✻ MARS Test |  | 16.475 |  | 1 |  | 16.475 |  | 1.552 |  | 0.215 |  | 0.011 |  |
| Stimulation Type ✻ Fluency Type ✻ Time ✻ Region ✻ MARS Test |  | 2.034 |  | 1 |  | 2.034 |  | 0.192 |  | 0.662 |  | 0.001 |  |
| Stimulation Type ✻ Fluency Type ✻ Time ✻ Region ✻ AGEGROUP |  | 1.128 |  | 1 |  | 1.128 |  | 0.106 |  | 0.745 |  | 7.696×10^-4^ |  |
| Residuals |  | 1464.811 |  | 138 |  | 10.615 |  |  |  |  |  |  |  |
|  | | | | | | | | | | | | | |
| *Note.*  Type III Sum of Squares | | | | | | | | | | | | | |

| **Between Subjects Effects** | | | | | | | | | | | | | |
| --- | --- | --- | --- | --- | --- | --- | --- | --- | --- | --- | --- | --- | --- |
| **Cases** | | **Sum of Squares** | | **df** | | **Mean Square** | | **F** | | **p** | | **η²_p_** | |
| **Region** |  | **1.100** |  | **1** |  | **1.100** |  | **0.016** |  | **0.898** |  | **1.194×10^-4^** |  |
| **AGEGROUP** |  | **776.090** |  | **1** |  | **776.090** |  | **11.631** |  | **< .001** |  | **0.078** |  |
| **MARS Test** |  | **1018.622** |  | **1** |  | **1018.622** |  | **15.265** |  | **< .001** |  | **0.100** |  |
| **Region ✻ MARS Test** |  | **0.010** |  | **1** |  | **0.010** |  | **1.517×10^-4^** |  | **0.990** |  | **1.099×10^-6^** |  |
| **Region ✻ AGEGROUP** |  | **24.165** |  | **1** |  | **24.165** |  | **0.362** |  | **0.548** |  | **0.003** |  |
| **Residuals** |  | **9208.498** |  | **138** |  | **66.728** |  |  |  |  |  |  |  |
|  | | | | | | | | | | | | | |
| ***Note.*  Type III Sum of Squares** | | | | | | | | | | | | | |

**Descriptives**

| **Descriptives** | | | | | | | | | | | | | | | | | | | |
| --- | --- | --- | --- | --- | --- | --- | --- | --- | --- | --- | --- | --- | --- | --- | --- | --- | --- | --- | --- |
| **Stimulation Type** | | **Fluency Type** | | **Time** | | **Region** | | **AGEGROUP** | | **N** | | **Mean** | | **SD** | | **SE** | | **Coefficient of variation** | |
| Sham |  | Semantic |  | F15 |  | F |  | Older |  | 36 |  | 15.667 |  | 3.234 |  | 0.539 |  | 0.206 |  |
|  |  |  |  |  |  |  |  | Younger |  | 36 |  | 16.972 |  | 3.460 |  | 0.577 |  | 0.204 |  |
|  |  |  |  |  |  | T |  | Older |  | 36 |  | 15.583 |  | 3.237 |  | 0.540 |  | 0.208 |  |
|  |  |  |  |  |  |  |  | Younger |  | 36 |  | 16.194 |  | 2.896 |  | 0.483 |  | 0.179 |  |
|  |  |  |  | L45 |  | F |  | Older |  | 36 |  | 21.056 |  | 5.356 |  | 0.893 |  | 0.254 |  |
|  |  |  |  |  |  |  |  | Younger |  | 36 |  | 19.778 |  | 7.059 |  | 1.177 |  | 0.357 |  |
|  |  |  |  |  |  | T |  | Older |  | 36 |  | 20.194 |  | 5.549 |  | 0.925 |  | 0.275 |  |
|  |  |  |  |  |  |  |  | Younger |  | 36 |  | 20.972 |  | 5.639 |  | 0.940 |  | 0.269 |  |
|  |  | Phonemic |  | F15 |  | F |  | Older |  | 36 |  | 11.139 |  | 3.053 |  | 0.509 |  | 0.274 |  |
|  |  |  |  |  |  |  |  | Younger |  | 36 |  | 9.694 |  | 3.179 |  | 0.530 |  | 0.328 |  |
|  |  |  |  |  |  | T |  | Older |  | 36 |  | 10.000 |  | 2.918 |  | 0.486 |  | 0.292 |  |
|  |  |  |  |  |  |  |  | Younger |  | 36 |  | 9.667 |  | 3.610 |  | 0.602 |  | 0.373 |  |
|  |  |  |  | L45 |  | F |  | Older |  | 36 |  | 19.417 |  | 7.439 |  | 1.240 |  | 0.383 |  |
|  |  |  |  |  |  |  |  | Younger |  | 36 |  | 14.528 |  | 4.754 |  | 0.792 |  | 0.327 |  |
|  |  |  |  |  |  | T |  | Older |  | 36 |  | 17.333 |  | 5.550 |  | 0.925 |  | 0.320 |  |
|  |  |  |  |  |  |  |  | Younger |  | 36 |  | 13.944 |  | 5.722 |  | 0.954 |  | 0.410 |  |
| Anodal |  | Semantic |  | F15 |  | F |  | Older |  | 36 |  | 16.472 |  | 3.334 |  | 0.556 |  | 0.202 |  |
|  |  |  |  |  |  |  |  | Younger |  | 36 |  | 17.667 |  | 4.216 |  | 0.703 |  | 0.239 |  |
|  |  |  |  |  |  | T |  | Older |  | 36 |  | 15.556 |  | 3.367 |  | 0.561 |  | 0.216 |  |
|  |  |  |  |  |  |  |  | Younger |  | 36 |  | 16.389 |  | 2.821 |  | 0.470 |  | 0.172 |  |
|  |  |  |  | L45 |  | F |  | Older |  | 36 |  | 22.083 |  | 6.733 |  | 1.122 |  | 0.305 |  |
|  |  |  |  |  |  |  |  | Younger |  | 36 |  | 19.750 |  | 6.063 |  | 1.011 |  | 0.307 |  |
|  |  |  |  |  |  | T |  | Older |  | 36 |  | 21.028 |  | 7.033 |  | 1.172 |  | 0.334 |  |
|  |  |  |  |  |  |  |  | Younger |  | 36 |  | 20.167 |  | 6.349 |  | 1.058 |  | 0.315 |  |
|  |  | Phonemic |  | F15 |  | F |  | Older |  | 36 |  | 11.167 |  | 2.823 |  | 0.471 |  | 0.253 |  |
|  |  |  |  |  |  |  |  | Younger |  | 36 |  | 9.972 |  | 3.167 |  | 0.528 |  | 0.318 |  |
|  |  |  |  |  |  | T |  | Older |  | 36 |  | 10.861 |  | 2.664 |  | 0.444 |  | 0.245 |  |
|  |  |  |  |  |  |  |  | Younger |  | 36 |  | 9.083 |  | 2.999 |  | 0.500 |  | 0.330 |  |
|  |  |  |  | L45 |  | F |  | Older |  | 36 |  | 16.722 |  | 6.700 |  | 1.117 |  | 0.401 |  |
|  |  |  |  |  |  |  |  | Younger |  | 36 |  | 14.028 |  | 5.135 |  | 0.856 |  | 0.366 |  |
|  |  |  |  |  |  | T |  | Older |  | 36 |  | 18.194 |  | 4.387 |  | 0.731 |  | 0.241 |  |
|  |  |  |  |  |  |  |  | Younger |  | 36 |  | 15.889 |  | 5.371 |  | 0.895 |  | 0.338 |  |
|  | | | | | | | | | | | | | | | | | | | |
